# Link between the skin and autism spectrum disorder

**DOI:** 10.3389/fpsyt.2023.1265472

**Published:** 2023-10-18

**Authors:** Mao-Qiang Man, Shuyun Yang, Theodora M. Mauro, Guoqiang Zhang, Tingting Zhu

**Affiliations:** ^1^Dermatology Hospital, Southern Medical University, Guangzhou, China; ^2^Department of Dermatology, University of California, San Francisco, CA, United States; ^3^Dermatology Service, San Francisco VA Medical Center,San Francisco, CA, United States; ^4^Department of Dermatology, The People’s Hospital of Baoshan, Baoshan, China; ^5^Department of Dermatology, The First Hospital of Hebei Medical University, Shijiazhuang, China; ^6^Department of Dermatology, The First Affiliated Hospital of Soochow University, Suzhou, China

**Keywords:** epidermal permeability barrier, hydration, stratum corneum, autism, inflammation

## Abstract

Autism spectrum disorder (ASD) is a common neurological disorder. Although the etiologies of ASD have been widely speculated, evidence also supports the pathogenic role of cutaneous inflammation in autism. The prevalence of ASD is higher in individuals with inflammatory dermatoses than in those without inflammatory diseases. Anti-inflammation therapy alleviates symptoms of ASD. Recent studies suggest a link between epidermal dysfunction and ASD. In the murine model, mice with ASD display epidermal dysfunction, accompanied by increased expression levels of proinflammatory cytokines in both the skin and the brain. Children with ASD, which develops in their early lifetime, also exhibit altered epidermal function. Interestingly, improvement in epidermal function alleviates some symptoms of ASD. This line of evidence suggests a pathogenic role of cutaneous dysfunction in ASD. Either an improvement in epidermal function or effective treatment of inflammatory dermatoses can be an alternative approach to the management of ASD. We summarize here the current evidence of the association between the skin and ASD.

## 1. Introduction

Neurological disorders are common and can involve both children and adults with various prevalence, depending on geographic region, age, and gender. For instance, the prevalence of autism spectrum disorder (ASD), also termed autism, is approximately 2.21% in adults (1) and 1.4–2.8% in children aged 8 years in the United States (2, 3). In children, the prevalence of ASD increases with age, with a higher prevalence in boys (4.4% at the age of 10 years) than in girls (1.2% at the age of 10 years) (4). Similarly, a higher portion of adults with ASD are men (69.3% vs. 30.7%) (5). However, the gender differences in prevalence decline after age of 35 years (5). Moreover, a higher prevalence was observed in North America and high-income countries than in other regions and low-income countries (6). In adults, ASD is associated with intellectual, physical, and mental disabilities (5). Children with ASD display delayed mental development in multiple domains, with a negative correlation of developmental levels with disease severity (7). The annual medical costs for adult outpatients with ASD are five times of that for those without ASD ($4,375 vs. $824) (8). Average annual medical costs for children with ASD are nine times for those without ASD ($9,980 vs. $1102) (9). Thus, ASD is common and can negatively impact patients’ lives and economy.

ASD includes non-syndromic and syndromic autism. The latter is mainly caused by chromosomal abnormalities or monogenic alterations (10). Regarding the etiologies of non-syndromic ASD, it is still unclear yet. Although genetic and environmental factors can contribute to the development of ASD (11), evidence also indicates an association of ASD with immune function. For example, the prevalence of allergies and autoimmune diseases is higher in individuals with ASD than in those without ASD in both children and young adults (12–15). Epidemiological studies reveal a higher prevalence of ASD in individuals with inflammatory skin disorders, such as atopic dermatitis and psoriasis (12, 16). A recent study demonstrates epidermal dysfunction, which can provoke cutaneous and systemic inflammation, in children with ASD (17). In this perspective review, we summarize the evidence of a link between the skin and ASD and discuss the implication.

## 2. Link between dermatoses and ASD

The pathogenic role of inflammation in ASD has been well demonstrated (18, 19). Individuals with ASD display the upregulation of activator protein-1-mediated neuroinflammation and insulin/insulin-like growth factor-1 signaling pathways in the brain (20) although one study showed no significant differences in expression levels of either proinflammatory or anti-inflammatory genes, including IL-1β, in both the gray and white matter of the brain in normal vs. individuals with ASD (21). Moreover, the activation state of astrocytes is increased in both the gray and white matter of prefrontal areas in individuals with ASD, indicating the presence of chronic inflammation in the brain (22). Furthermore, circulating levels of some proinflammatory cytokines, such as IL-1β, IL-6, TNF-α, and IFN-γ, are also higher in individuals with ASD than in the controls (23–25), while circulating levels of anti-inflammatory cytokines, such as IL-10 and IL-1Ra, are decreased in individuals with ASD (26). Although this line of evidence suggests a pathogenic role of inflammation in ASD, the origins of inflammation are still inconclusive. Some studies demonstrate the contribution of inflammation associated with dysbiosis and/or with the increased gut permeability to the pathogenesis of ASD (26–30). Maternal immune activation is also a risk factor for ASD in offspring (31–33). However, a great bulk of evidence indicates a link between cutaneous inflammation to ASD. Moreover, the association of other cutaneous conditions with ASD has also been reported. The evidence of the link between the skin and ASD is summarized in Table 1 (12, 16, 34–67).

**Table 1:** Association of autism with cutaneous function.

Cutaneous conditions/subjects	Association with autism spectrum disorder (ASD)	References
**Psoriasis**		
3–26 years old; 5,565 cases with autism (1,102 female and 4,563 male patients) and 27,825 controls (5,010 female and 22,815 male patients)	Prevalence of psoriasis is higher in children with ASD than in the controls (0.34% vs. 0.15%; OR = 2.35, 95% CI 1.36–4.08)	(12)
2–5 years old; 663 cases of ASD (117 female and 546 male patients) and 915 controls (419 female and 496 male patients)	81 cases of ASD (13.42%) with a family history of psoriasis/eczema; 95 controls (10.38%) with a family history of psoriasis/eczema. *p* = 0.07	(34)
Analyses of 708,517 families	Neither paternal nor maternal psoriasis increases the risk of ASD for their offspring	(35)
1,386,260 children aged 9.7 ± 2.3 years	A family history of psoriasis is not a risk factor for ASD	(36)
**Eczematous dermatitis**		
42,641 children and adults with AD and 71,699 controls	AD vs. Non-AD: OR = 1.24; 95% CI = 0.94 ~ 1.64; Mil AD: OR = 1.07, 95% CI = 0.66–1.74; Moderate AD: OR = 0.96, 95% CI = 0.33–2.84; Severe AD: OR = 2.54, 95% CI = 1.54–4.21, *p* = 0.0003	(37)
Analysis of data from 186 countries	No correlation between AD and ASD	(38)
Children with and without AD each had 387,262 cases. AD was diagnosed at the age of <2 years	Children with AD at age < 2 years have higher risk of ASD than that without AD (adjusted HR = 1.09, 95% CI = 1.02–1.17, *p* < 0.05)	(39)
18,473 children with AD aged 1 month to 3 years were enrolled and followed up for 3 years. 18,473 children without AD served as controls	Prevalence of ASD is higher in children with AD than the controls (0.99% vs. 0.09%, *p* < 0.001). (HR = 8.90, 95% CI = 4.98–15.92)	(40)
8,130 children with AD were enrolled at birth and followed up for 10–13 years. 6,944 children without AD served as controls	Prevalence of ASD is higher in children with AD than in the controls (0.8% vs. 0.2%, *p* < 0.0001). (HR = 3.4, 95% CI = 1.95–5.93)	(41)
25,691 children with AD and 22,713 without AD, aged 2–17 years	Prevalence of ASD is higher in children with AD than in the controls (1.9% vs. 0.9%). (aOR = 1.52, 95% CI = 1.29–1.78, *p* < 0.001)	(42)
3,911 ASD cases and 38,609 controls aged 2–12 years	Children with ASD have slightly high prevalence of AD than the controls (21.09% vs. 18.85%; aOR = 1.1, 95% CI = 1.01–1.20)	(43)
79,667 children aged 0–18 years were analyzed	Prevalence of ASD is higher in children with eczema than in the controls (2.19% vs. 0.89%, *p* < 0.0001). (OR = 2.51, 95% CI = 1.67–3.77)	(44)
Phone interview of parents of 27,566 children aged 0–5 years	Prevalence of eczema is higher in children with ASD than in the controls (28.4% vs. 15.4%, aOR = 7.17, 95% CI = 2.56–20.04)	(45)
41,244 children aged 3–17 years	Prevalence of eczema is higher in children with ASD than in the controls (15.6% vs. 10.2%, aOR = 1.7, 95% CI = 1.2–2.5)	(46)
341 children aged 3–6 years	Children with higher SCQ score (>11) have a higher prevalence of eczema symptoms than those with lower SCQ score (33.3% vs. 10.4%, *p* < 0.0001)	(47)
1,596 children with ASD and 6,384 controls	Prevalence of AD is higher in children with ASD than in the controls (17.8% vs. 13.0%, *p* < 0.001; OR = 1.52, 95% CI = 1.30–1.78)	(48)
578 children with ASD and 2,312 controls	Children with ASD have a higher prevalence of AD than the controls (17.3% vs. 11.3%, *p* < 0.001; OR = 1.24, 95% CI 1.04–1.48)	(49)
324,000 children with ASD and 61,100,000 without ASD, aged 3–17 years	Children with ASD have a higher prevalence of eczema than the controls (14.9% vs. 9.2%; OR = 1.4, 95% CI =1.0–2.1)	(50)
2,762 cases with ASD-affected siblings and 11,048 controls	Individuals with ASD -affected siblings had higher incidence of AD (9.9% vs. 8.1%, *p* = 0.003) with relative risk of 1.22 (95% CI 1.09–1.36)	(51)
45 atopic (AD >55%) and 93 non-atopic children (mean age 6 years) with autism	Children with atopic condition have higher ADOS CSS scores on total scale than those without atopic condition (7.79 ± 1.51 vs. 7.16 ± 1.86, *p* = 0.039). ADOS CSS mean scores in the RRB symptom domain do not differ significantly between children with vs. without atopic condition (7.42 ± 1.94 vs. 7.45 ± 2.22, *p* = 0.943)	(16)
Children aged 3–26 years, including 5,565 children with ASD and 27,825 controls	Prevalence of dermatitis is comparable in children with vs. without ASD (7.3% vs. 6.9%; OR = 1.07, 95% CI = 0.95–1.20)	(12)
2,580 children were enrolled at birth and followed up for 2 to 18 years	ASD is not associated with AD (adjust relative risk ratio = 1.38, *p* = 0.121)	(52)
26 children with ASD and 107 controls	Prevalence of ASD does not differ significantly between children with or without AD (3.8% vs. 5.6%)	(53)
24,938 children aged <3 years	Children with AD have a higher risk for ASD (aHR = 1.568, 95% CI = 1.109–2.218, *p* = 0.011)	(54)
234,170 children aged 2–18 years	Children with eczematous dermatitis have a higher risk for ASD (aHR = 1.4, 95% CI = 1.28–1.54, *p* < 0.0001)	(55)
1,386,260 children aged 9.7 ± 2.3 years	A family member with AD increases the risk for ASD (aHR = 1.29, aHR = 1.25–1.33, *p* < 0.001)	(36)
**Pigmented skin disorders**		
A 13-year-old boy born with oculocutaneous albinism had symptoms of ASD on growing up		(56)
Three families with ASD and oculocutaneous albinism		(57)
Two boys with ASD and oculocutaneous albinism		(58)
Two siblings with ASD and oculocutaneous albinism		(59)
In two families, each has a twin with ASD and hypomelanosis of Ito		(60)
Two girls and a boy have both ASD and hypomelanosis of Ito		(61)
Prevalence of hyperpigmented skin disorders is higher in children with ASD than in those without ASD (56.8% vs. 32.6%, OR = 3.12, *p* = 0.0001). Pigmentary mosaicism of hyperpigmented type (OR = 2.76, *p* = 0.02); Café-au-lait macules (CALMs) (OR = 2.40, *p* = 0.001)		(62)
**X-linked ichthyosis**		
Five boys with X-linked ichthyosis also have ASD		(63)
A boy has both X-linked ichthyosis and ASD		(64)
3 out of 58 adults and 4 out of 24 children with X-linked ichthyosis have ASD		(65)
1% (1/94) of female carriers of genetic mutations associated with X-linked ichthyosis have ASD		(66)
A boy with X-linked ichthyosis has ASD		(67)

OR, odds ratio; aOR, adjusted odds ratio; HR, hazard ratio; aHR, adjusted hazard ratio; ADOS, Autism Diagnostic Observation Schedule; CSS, calibrated severity scores; CSS-SA, subdomains of social affect; CSS-RRB, restricted and repetitive behavior; AD, atopic dermatitis; SCQ, Social Communication Questionnaire.

### 2.1. Psoriasis

Psoriasis, a chronic inflammatory disorder, is linked to a number of extracutaneous comorbidities, such as cardiovascular diseases, diabetes, and obesity (68, 69). Studies also show a link between psoriasis and ASD. For example, the prevalence of psoriasis in children with ASD is twice as that in children without ASD (0.34% vs. 0.15%, OR = 2.35, 95% CI = 1.36–4.08) (12). Interestingly, a family history of psoriasis is also a risk factor for ASD for offspring (aHR = 1.55, 95% CI = 1.07–2.23, *p* = 0.019) (36). Although one study did not show the link between ADS and maternal immune conditions, such as psoriasis, in children (70), Croen et al. reported that 13.4% of individuals with ASD have a maternal history of psoriasis (34). The same group conducted another study in 407 cases of ASD and 2095 controls. The results showed that the mothers of 2.7% of children with ASD had psoriasis at the time of pregnancy, while the mothers of 1% of children with ASD had psoriasis in the controls (aOR = 2.7, 95% CI = 1.3–5.8, *p* = 0.003) (71). This phenomenon is likely due to the inflammation in the brain of offspring, induced by IL-17A, a key cytokine in psoriasis, from the mother because injection of IL-17A into fetal lateral ventricle can induce ASD-like phenotype in mice (72). The link between psoriasis and ASD is further supported by several observations in mice and humans. First, circulating levels of IL-17 are elevated in subjects with either psoriasis or ASD (including murine models) (73–75). Similarly, increased levels of IL-17A have also been demonstrated in the brain of mice with ASD (76). Second, serum IL-17A levels are positively correlated with the severity of ASD in humans (74). The inhibition of IL-17A signaling improves ASD symptoms in a mouse model of ASD (77). Similarly, the treatment of psoriasis with Secukinumab, an IL-17A inhibitor, also alleviates psychological symptoms of ASD in humans (78). Collectively, this line of evidence indicates a link between ASD and psoriasis.

### 2.2. Eczematous dermatitis

Eczematous dermatitis, another common inflammatory skin condition, exhibits elevated circulating levels of cytokines, including IL-17 (79, 80), which has been linked to the development of ASD (72, 81). Several epidemiological studies demonstrate an association of eczematous dermatitis with ASD. For example, up to 25% of children with ASD had atopic dermatitis (82, 83). Similarly, a study in a large cohort showed that the prevalence of eczema is higher in children with ASD than in the controls (28.4% vs. 15.4%, aOR = 7.17, 95% CI = 2.56–20.04) (45). Similarly, more than 52% of individuals with ASD have allergic manifestations (including atopic dermatitis) (vs. 10% in subjects without ASD, *p* < 0.001) (84). Interestingly, children with ASD-affected siblings have a higher risk for atopic dermatitis with a relative risk ratio of 1.22 (95% CI = 1.09–1.36) (incidence 9.9% vs. 8.1%, *p* = 0.003) (51). On the other hand, the prevalence of ASD is higher in children with atopic dermatitis than in the controls in Chinese (0.99% vs. 0.09%, *p* < 0.001; HR = 8.90, 95% CI = 4.98–15.92) (40). Another study conducted in the US showed that the prevalence of ASD in children with atopic dermatitis was twice of that in children without atopic dermatitis (1.9% vs. 0.9%, aOR = 1.52, 95% CI = 1.29–1.78, *p* < 0.001) (42). Atopic dermatitis does not only increase the risk for ASD but also increases the severity of ASD. The Autism Diagnostic Observation Schedule Calibrated Severity scores are higher in children with both ASD and atopic dermatitis than in those with ASD alone (*p* < 0.05) (16). However, some studies did not show differences in the prevalence of ASD between children with vs. without atopic dermatitis (12, 52, 53). The discrepant results in the association of ASD with atopic dermatitis could be due to the severity of atopic dermatitis in the individuals because only children with severe atopic dermatitis are associated with the risk for ASD (OR = 2.54, 95% CI = 1.54–42.1, *p* = 0.003) (37). But odds ratios of ASD in individuals with mild and moderate atopic dermatitis are 1.07 (95% CI = 0.66–1.74) and 0.96 (95% CI = 0.33–2.84), respectively (37). Additionally, mothers who have atopic eczema at the time of pregnancy increase the risk of ASD for offspring (aOR = 1.8, 95% CI = 1.0–3.4, *p* = 0.04) (66). Taken together, this line of evidence demonstrates a link between ASD and eczematous dermatitis.

### 2.3. Other skin disorders

In addition to inflammatory dermatoses, other cutaneous conditions are also associated with ASD. There are several reports of an association between hypomelanotic disorders and ASD (56–61). For example, linear and whorled hypermelanosis complicated with ASD has also been reported (85, 86). Studies showed that up to 60% of individuals with hypomelanotic disorders have ASD [reviewed in (87)]. It has been postulated that vitamin D deficiency contributes to the pathogenesis of ASD in hypomelanotic disorders. This theory could hold true to some extent. Epidemiological studies showed that lower levels of maternal vitamin D increase the risk of ASD for offspring (88) and that children with ASD have lower levels of serum vitamin D (89). Moreover, because of the sunburn concern, individuals with hypomelanotic disorders may try to avoid sun exposure, resulting in a reduction in endogenous production of active vitamin D in the body. Vitamin deficiency has been linked to inflammatory disorders (90). This assumption is also supported by several observations: (a) serum vitamin D levels are negatively correlated with levels of inflammatory markers (C-reactive protein, calprotectin, and fibrinogen) and disease severity in individuals with either Crohn’s disease or ulcerative colitis (91); (b) supplement of vitamin D lowers levels of proinflammatory cytokines *in vitro* and *in vivo* (92, 93). However, hyperpigmented skin disorder can also be complicated with ASD. Pinheiro et al. reported that a 5-year-old boy with hyperpigmentation along the lines of Blaschko was complicated with ASD (94). Thus, further studies are needed to elucidate the underlying mechanisms by which pigmented skin disorders are linked to ASD. Other cutaneous conditions, such as alopecia areata and X-linked ichthyosis, can also be complicated with ASD. For instance, the prevalence of alopecia areata in individuals with ASD is higher than in that without alopecia areata (13.61% vs. 10.43%, HR = 1.238, 95% CI = 1.100–1.395) (95). There are also several case reports of ASD complicated with X-linked ichthyosis or microphthalmia with linear skin defects syndrome (63–67, 96). Nevertheless, these multiple lines of evidence indicate a link between skin disorders and ASD.

In addition to skin disorders, stimulation of the skin with deep pressure alleviates physiological responses, including decreased heart rate and skin conductance, in children with ASD (97, 98). However, the deep pressure-induced changes in physiological function are likely attributable to the altered neural function via efferent pathways and sudomotor impulses, resulting from the discomfort caused by deep pressure (97, 99). Moreover, the contact of the skin of the subject can also influence the symptoms of ASD, depending on the sensory responses. For example, hypo-responsiveness, but not hyper-responsiveness, to tactile stimuli is associated with aberrant social and communication (100). Hence, the skin is also associated with ASD in individuals without dermatoses.

## 3. Association of epidermal dysfunction and ASD

As aforementioned, ASD is associated with a number of skin disorders. Recent studies also demonstrated alterations in epidermal function in ASD. In the murine model of ASD, mice with ASD exhibit dry skin and a remarkable increase in transepidermal water loss rates, an indicator of epidermal permeability barrier function, compared to normal mice (76). Similarly, adult humans with ASD also display elevated transepidermal water loss rates (76). Recently, Wang et al. compared epidermal biophysical properties in 48 control children and 56 children with ASD (17). All participants were without a current or previous history of inflammatory skin disorders. Children with ASD exhibited higher transepidermal water loss rates, lower stratum corneum hydration levels, and elevated skin surface pH (all *p* < 0.0001 vs. controls), indicating epidermal dysfunction in ASD. Most importantly, the ASD symptom such as the Social Responsiveness Scale was significantly improved following the correction of epidermal functional abnormalities with topical emollient (CURECODE^®^) twice daily for 2 months (146.40 ± 4.83 vs. 136.8 ± 5.70, *t* = 2.988, *p* < 0.01). The autism treatment evaluation checklist and repetitive behaviors scale-revised were also markedly reduced (*p* = 0.0777 and *p* = 0.0621, respectively) (101). These results suggest that ASD is also linked to epidermal dysfunction.

## 4. Perspectives

ASD is a common neurological disorder with a worldwide prevalence of as high as 4.36% (102). The etiologies of ASD have been widely speculated. Evidence suggests that ASD can be attributed to genetic, epigenetic, and environmental factors in addition to microbiome (10, 103–105). Indeed, a family history of ASD increases the odds of ASD in their children by 7.4–16.2 times (106). Somatic variants account for 3–5% of ASD (107, 108). If one has ASD, the other one is more likely to have it in monozygotic twins (109). Epigenetic modifications, such as DNA methylation and histone modification, also contribute to the development of ASD (105). Moreover, several studies demonstrate the role of gut microbiota in the pathogenesis of ASD (110, 111). Children with ASD have a high incidence of gastrointestinal disorders, such as constipation and diarrhea (112). Children with constipation have a higher risk of ASD (adjusted hazard ratio = 1.431, 95% CI = 1.083–1.891) (54). Both gut microbiota and their metabolic products are altered in humans and animals with ASD (113–115). In comparison to the non-ASD controls, children with ASD have higher fecal levels of Caloramator, Sarcina, and Clostridium genera and lower levels of Eubacteriaceae (116). The pathogenic role of gut microbiota in ASD is further supported by the evidence that oral probiotics during pregnancy prevent both the development of ASD symptoms and the increases in IL-6 and IL-17a levels in the brain of offspring induced by maternal immune activation (117). Similarly, orally given human commensal *bacteroides fragilis* alleviates ASD symptoms, improves gut permeability, and alters microbial composition in a mouse model of ASD induced by maternal immune activation (118). Apparently, the pathogenic role of microbiota in ASD is mediated by inflammation. The structural components of the bacteria can induce the production of proinflammatory cytokines, including IL-1β, IL-6, and TNF-α (110, 119). Individuals with ASD exhibit high intestinal permeability, resulting in an increased antigenic load from the gastrointestinal tract (113). Similarly, the brain–blood barrier is also compromised in individuals with ASD (120). Cytokines, such as IL-1β, IL-6, IFN-γ, and TNF-α, released from the intestine can easily penetrate into the circulation and cross the blood–brain barrier into the brain, consequently leading to the development of ASD (110). In support of the inflammation theory, individuals with ASD display higher levels of proinflammatory cytokines in both circulation and the brain (74, 76, 117, 121), while circulating levels of anti-inflammatory cytokines, such as IL-10 and IL-1Ra, are lower in individuals with ASD vs. normal controls (122). Accordingly, an anti-inflammation regimen improves symptoms of ASD (123). Thus, inflammation plays a central role in the development of ASD.

Consistent with the notion of the central role of inflammation in ASD, inflammatory dermatoses, such as psoriasis and eczematous dermatitis, predispose to the development of ASD. First, the development of ASD occurs approximately 6 months following the first appearance of allergic disorders in children (55). Second, in the murine model of ASD, increased cytokine levels in the brain are later than those in the skin (76). Third, the treatment of psoriasis with Secukinumab improves the symptoms of ASD (78). Coupling with the evidence of a positive correlation of the severity of ASD symptoms with the severity of atopic dermatitis, cytokines of cutaneous origin can also contribute to the development of ASD. However, a maximum of 28% of individuals with ASD have atopic dermatitis (45). Not all individuals with ASD have gastrointestinal disorders. Then, the question is where the inflammation is in individuals without either gastrointestinal disorders or inflammatory skin diseases. A bulk of evidence suggests a possible contribution of epidermal dysfunction to inflammation in both circulation and the brain. First, children with ASD, but without allergic disorders nor inflammatory dermatoses, display alterations in epidermal biophysical properties, including elevations in transepidermal water loss rates and skin surface pH and reduction in stratum corneum hydration levels (17). Second, elevation in transepidermal water loss rates increases levels of proinflammatory cytokines in both circulation and the skin (124). Third, reduction in stratum corneum hydration levels increases cutaneous inflammation (125–127), while circulating levels of proinflammatory cytokines are negatively correlated with stratum corneum hydration levels in humans without apparent inflammatory cutaneous inflammation (128). Because of the huge surface area of the skin (2 m^2^ for adult males and 1.7 m^2^ for adult females) (129), even mild cutaneous inflammation induced by epidermal dysfunction can increase proinflammatory cytokines in circulation. Elevated circulating levels of proinflammatory cytokines downregulate expression levels of a tight junction protein (occludin 1), as well as vascular cell adhesion molecule 1 in the endothelial cells of the brain, leading to an increase in blood–brain permeability, subsequently resulting in inflammation in the brain (130). Finally, improvement in epidermal function lowers circulating levels of proinflammatory cytokines in both humans and mice (124, 131) and alleviates some symptoms of ASD in children without any inflammatory dermatoses in addition to mitigation of other psychological symptoms such as cognitive impairment in adults (101, 132). Hence, this pile of evidence indicates that the development of ASD can be attributable, at least in part, to epidermal dysfunction. Because of the regulatory role of epidermal function in cutaneous inflammation, improvement in epidermal function can benefit ASD.

## 5. Conclusion

ASD is common in both adults and children. Although therapeutic regimens, such as medication and behavior management, are available, there are still challenges in clinical practice. Medicines, such as risperidone and aripiprazole, can cause some side effects, including somnolence, extra-pyramidal symptoms, hyperprolactinemia, body weight gain, headache, and urinary incontinence. (133, 134). Moreover, either medication or behavior management can only improve symptoms without solving the fundamental problem, inflammation, a key contributor to the pathogenesis of ASD. However, recent studies showed that an improvement in epidermal function with topical emollients can lower cytokine levels in both the skin and circulation of mice and humans (124, 131). Similarly, topical emollients can both improve and prevent the development of inflammatory skin disorders, including atopic dermatitis and psoriasis (135–139), which both are associated with ASD, as mentioned above. Taken together with the evidence that topical emollients improve symptoms of ASD and mitigate the progression of cognitive impairment, which both are linked to inflammation, either improvement in epidermal function with emollients, especially in individuals with epidermal dysfunction or effective treatments of inflammatory dermatoses can be an alternative approach in the management of ASD. However, this speculation needs to be validated in appropriate clinical trials.

## Data availability statement

The original contributions presented in the study are included in the article/supplementary material, further inquiries can be directed to the corresponding authors.

## Author contributions

M-QM: Conceptualization, Writing – original draft, Writing – review & editing. SY: Data curation, Formal analysis, Investigation, Writing – original draft. TM: Writing – review & editing. GZ: Conceptualization, Data curation, Formal analysis, Writing – review & editing. TZ: Writing – review & editing, Conceptualization, Data curation.
